# The use of the Airtraq^® ^optical laryngoscope for routine tracheal intubation in high-risk cardio-surgical patients

**DOI:** 10.1186/1756-0500-4-425

**Published:** 2011-10-19

**Authors:** Gereon Schälte, Ulrike Scheid, Steffen Rex, Mark Coburn, Britta Fiedler, Rolf Rossaint, Norbert Zoremba

**Affiliations:** 1 Department of Anesthesiology, University Hospital Aachen, Aachen, Germany

## Abstract

**Background:**

The Airtraq^® ^optical laryngoscope (Prodol Ltd., Vizcaya, Spain) is a novel disposable device facilitating tracheal intubation in routine and difficult airway patients. No data investigating routine tracheal intubation using the Airtaq^® ^in patients at a high cardiac risk are available at present. Purpose of this study was to investigate the feasibility and hemodynamic implications of tracheal intubation with the Aitraq^® ^optical laryngoscope, in high-risk cardio-surgical patients.

**Methods:**

123 consecutive ASA III patients undergoing elective coronary artery bypass grafting were routinely intubated with the Airtraq^® ^laryngoscope. Induction of anesthesia was standardized according to our institutional protocol. All tracheal intubations were performed by six anesthetists trained in the use of the Airtraq^® ^prior.

**Results:**

Overall success rate was 100% (n = 123). All but five patients trachea could be intubated in the first attempt (95,9%). 5 patients were intubated in a 2nd (n = 4) or 3rd (n = 1) attempt. Mean intubation time was 24.3 s (range 16-128 s). Heart rate, arterial blood pressure and SpO_2 _were not significantly altered. Minor complications were observed in 6 patients (4,8%), i.e. two lesions of the lips and four minor superficial mucosal bleedings. Intubation duration (p = 0.62) and number of attempts (p = 0.26) were independent from BMI and Mallampati score.

**Conclusion:**

Tracheal intubation with the Airtraq^® ^optical laryngoscope was feasible, save and easy to perform in high-risk patients undergoing cardiac surgery. In all patients, a sufficient view on the vocal cords could be obtained, independent from BMI and preoperative Mallampati score.

**Trial Registration:**

DRKS 00003230

## Background

Tracheal intubation is still the golden standard of securing the airway under clinical and preclinical conditions. In case of resuscitation, respiratory failure, unconsciousness and loss of a patent airway it is life saving. Since its introduction in 1941, the Macintosh laryngoscope has been the most popular device used for intubation worldwide.

However, tracheal intubation using this laryngoscope has been demonstrated to fail in up to 35% of patients with an unpredicted difficult airway [1,2]. Problems in securing the airway are still the main contributors to anesthesia-related morbidity and mortality [3]. Therefore, a wide variety of alternatives to the Macintosh laryngoscope have been introduced into clinical routine, including the Miller-, McCoy-, and Bullard-laryngoscope. Since 1982 Archie Brain's supra glottic laryngeal mask airway (LMA) and subsequently introduced diverse supra glottic airway devices, (laryngeal tube (LT), the intubating laryngeal mask airway (ILMA)) revolutionized the management of the difficult airway [4,5]. Some of the devices enable guided blind, video- or fiberoptic guided tracheal intubation, such as the ILMA [6]. In the last decade indirect, video assisted laryngoscopy became most popular, allowing a better visualization of the anatomical landmarks and an improved teaching and learning curve [7-10]. The Airtraq^® ^optical laryngoscope (Prodol S.A., Vizcaya, Spain) is a single-use rigid video laryngoscope that has been developed to facilitate tracheal intubation in both, patients with normal or difficult to intubate airways [11-13]. The resulting glottic view is provided without an alignment of the oral, pharyngeal and tracheal axes. Airtraq^® ^laryngoscopes for nasal intubation, pediatric applications and double lumen tube intubation are available. The device can be completed with a wireless clip-on camera for external broadcast and teaching purposes.

Several trials in both simulators and in clinical practice demonstrated the Airtraq to be easier to use in routine airways, compared to the Macintosh Laryngoscope [11,14]. Managing the unexpected or expected difficult airway in adult and pediatric patients the Airtraq^® ^[13] has been reported to be an excellent and fast alternative device, even in rare syndromes [15-17].

Cardiac patients are more prone to develop hemodynamic instability on induction of anesthesia and frequently respond to stress with an increase of blood pressure and heart rate. This might lead to cardiac arrhythmia and myocardial ischemia [18,19]. Few studies in non-cardiac patients indicate the Airtraq^® ^to provoke more hemodynamic stability subsequent to the endotracheal intubation procedure and minor trauma as compared to the Macintosh laryngoscope [14].

Purpose of our study was to investigate the feasibility and hemodynamic implications of tracheal intubation with the Airtraq^® ^optical laryngoscope, in high-risk cardio-surgical patients scheduled for routine coronary artery bypass grafting. Potential side effects and complications using the Airtraq^® ^optical laryngoscope as the standard laryngoscope for tracheal intubation were studied.

## Methods

After approval by the institutional ethical board (RWTH Aachen University, Medical Faculty Ethical Committee, EK 117/11), waiving written informed consent, 123 consecutive ASA III patients scheduled for elective coronary artery bypass grafting were observed. Exclusion criteria were emergency procedures, a history of difficult intubation, an inter-incisor distance < 40 mm, reduced neck mobility and a Mallampati score of IV.

All patients received general anesthesia according to our institutional regimen. 90 min before induction of anesthesia, patients were pre-medicated with midazolam (3.75-7.5 mg p.o.). Standard monitoring including 6 lead ECG, pulse oximetry, non-invasive and invasive arterial blood pressure measurements was established prior to induction. After 2 min of pre-oxygenation (100% oxygen, 8 L*min^-1^), 0.25-0.5 μg*kg^-1 ^Sufentanil were given i.v.. Anesthesia was induced with 0.1-0.2 mg*kg^-1 ^Etomidate after additional 3 min of spontaneous or assisted bag valve ventilation. The patients' lungs were manually ventilated with 100% of oxygen and after achieving apnoe and Rocuronium 0.6 mg*kg^-1 ^was administered. After additional bag valve ventilation for another 2 min, the patients' trachea was intubated using the Airtraq^® ^optical laryngoscope. Mechanical ventilation was initiated (FiO_2 _0.5, V_T _6-8 ml*kg^-1^, respiratory rate 12-16 min^-1^) after completion of intubation. Anesthesia was maintained using a continuous infusion of sufentanil (0.3-0.6 μg*kg^-1^*h^-1^) and Sevoflurane (0.8-1.5%). All anesthetists engaged in this trial had been trained in the use of the Airtraq^® ^in manikin and had performed at least 6 successful clinical intubations in non-cardiac surgical patients prior to the investigation. For security reasons each intubation was supervised by an experienced Airtraq^® ^instructor (BF, GS).

In case of primary intubation failure, bag valve ventilation was re-established and, after another period of mask ventilation, a second intubation attempt was performed. In case of another futile attempt, or failure of tracheal intubation after a total of 120 s, the supervisor took over respectively and, after another 2 minutes of bag valve ventilation, performed one more attempt using the Airtraq^® ^. In case of futility, the institutional (ASA protocol derived) guidelines for the management of the difficult airway would have been applied. This modus operandi is in analogy to any induction of anesthesia performed in our hospital and includes fiberoptic bronchoscopy and/or the use of the intubation laryngeal mask, in case of definite failure.

Intubation time was recorded starting with passing the Airtraq^® ^through the lips and stopping once the lungs have successfully been ventilated. Moreover, success rates, SpO_2_, the quality of glottis visualization, common problems and side effects, e.g. soft tissue bleeding, laceration of the lips and contamination of the optical system were evaluated directly after the intubation and in the course of the operation. Acknowledging the limitations-in lack of a validated score describing glottis visualization whenever using modern video-laryngoscopes-to assess the ease of intubation and glottic view a graded intubation score with four endpoints equal to the Cormack-Lehane score was used [intubation score]. Scoring was performed under direct laryngoscopy with the Airtraq^® ^device and without the use of the videoscope monitor. Hemodynamic data and SpO_2 _were recorded at 4 different time points, on admission to the operation room (T1), before intubation (T2), one minute (T3) and five minutes after (T4) laryngoscopy and intubation.

### Statistical analysis

Statistical analysis was conducted using Prism 5 (version 5.0 for Mac OS X, Copyright^© ^1994-2009, the GraphPad Software, Inc.). Results are presented as means ± standard deviation (M ± SD) for continuous variables. Parameters were compared using ANOVA for repeated measurements with Bonferroni's correction for multiple comparisons. Spearman's correlation coefficient was calculated to describe relation between variables. Comparisons were considered statistically significant when p < 0.05.

## Results

From January 2011 to June 2011, 123 patients (64 male, 59 female) undergoing coronary artery bypass grafting were included in this observational feasibility trial. In all patients tracheal intubation with the Airtraq^® ^optical laryngoscope was feasible and safe. The participating anesthetists subjectively judged the intubation conditions as very good in 120 patients (97.6%) and as good in 3 patients (2.4%) (Table 1).

**Table 1:** Patient characteristics.

	Airtraq (n = 123)
Gender; male/female	64/59
Age; year	64.43 ± 15.41
Height; cm	171.6 ± 9.21
Weight; kg	82.85 ± 15.16
BMI; kg.m^-2^	28.15 ± 4.93
ASA	3

Demographical data and patient's characteristics. Data are numbers, means and standard deviation.

118 Patients (95.9%) were intubated at the first attempt. A second and a third attempt were required in four (3.2%) and one (0.8%) patients respectively. In all patients visualization of the vocal cords was very good. Based on the intubation-score, vocal cord view was judged as "Grade I" in 114 patients (92.7%) and Grade II in 9 patients (6.3%) (Table 2).

**Table 2:** Success rate, scores and judgment, n = 123

	I	II	III	IV
Intubation attempts, number	118	4	1	0
Cormack-Lehane Score	114	9	0	0
Conditions	120	3	0	0

Conditions = subjective judgment: I = excellent, II = good, III = moderate, IV = bad. Data are means +/- standard deviation.

**Figure 1:**
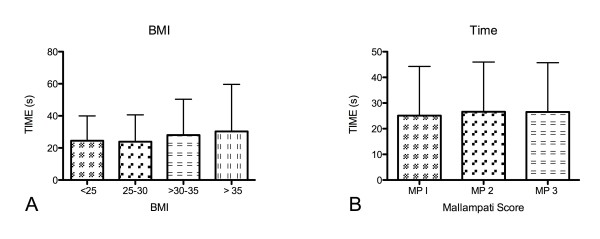
**The duration of intubation did not correlate with the BMI (p = 0.34) (Figure 1A) or the Mallampati score (p = 0.14) (Figure 1B)**. Data are presented as means ± standard deviation.

Time for tracheal intubation was 25.6 ± 19.1 s (range: 12 s-118 s). The duration of intubation did not correlate with the BMI (p = 0.34) (Figure 1A) or the Mallampati score (p = 0.14) (Figure 1B).

**Figure 2:**
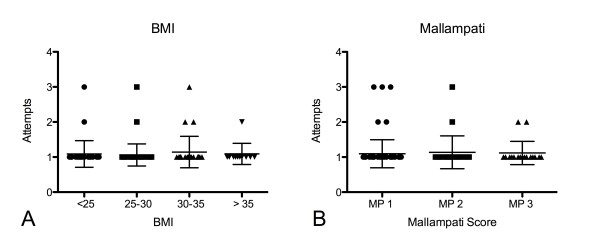
**No significant correlation between the number of attempts and the BMI (P = 0.70) (Figure 2A) or the Mallampati score (P = 0.69) (Figure 2B) could be shown**. Data are presented as means ± standard deviation.

Likewise, there was no correlation between the number of attempts and the BMI (P = 0.70) (Figure 2A) or the Mallampati score (P = 0.69) (Figure 2B).

In 3 of the primarily failed intubation attempts, the 2^nd ^and 3^rd ^attempt of laryngoscopy were necessary due to a drift of the endotracheal tube in the esophageal direction, despite an excellent view on the glottis and the upper esophageal aperture. Intubation was then successful after either the device position correction or reinsertion in a more ventral position and cranial position. One patient needed a BURP-maneuver (backward-upward-rightward-pressure on the larynx, performed manually to improve intubation conditions) to enable the correct placement of the endotracheal tube finally.

On account of their coronary artery disease these patients were medicated with aspirin and clopidogrel. 59% of our patients were treated with aspirin, 17% with aspirin and clopidogrel. In 24% all platelet aggregation inhibitors had been stopped 7 days before the operation. All patients were medicated with low molecular weight heparin.

We report about 6 minor complications (4.8%) during the intubation procedure: 4 minor mucosal damages and 2 minimal lip contusions. In two cases of mucosal damage previous antithrombotic therapy (aspirin and clopidogrel) led to a 2^nd ^intubation attempt due to a contamination of the optical system and required cleaning and oral suctioning. There was no incidence of dental trauma.

**Figure 3:**
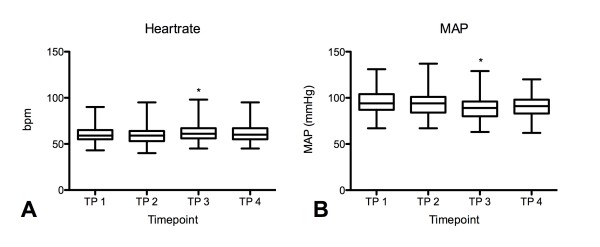
**At T3-one minute after intubation-heart rate increased (P < 0.029) (Figure 3A) and ABP decreased (P < 0.001) (Figure 3B)**. Both changes were judged clinically irrelevant. Five minutes after tracheal intubation (T4) no more differences to the baseline values could be detected (n.s.). Data are presented as means ± standard deviation.

Tracheal intubation with the Airtraq^® ^resulted in a minimal but significant increase of the heart rate (p < 0.029) (Figure 3A) and decrease of mean arterial blood pressure [MAP] (p < 0.001) (Figure 3B) one minute after intubation (T3). At T4, 5 min after intubation, no significant changes in heart rate and MAP compared to the baseline values could be detected.

**Figure 4:**
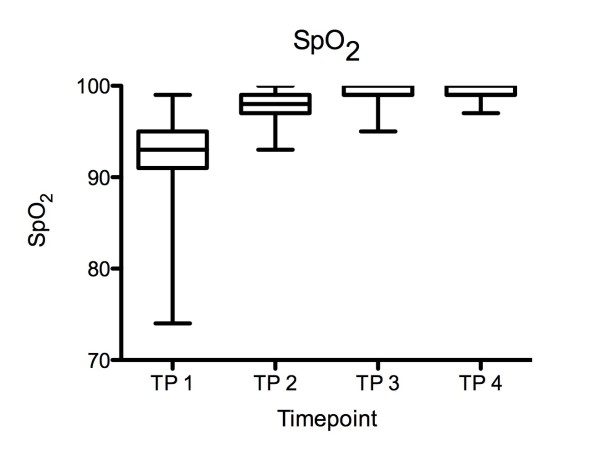
**Oxygen saturation [SpO_2_] during tracheal intubation: After initial pre-oxygenation and bag valve ventilation (T2), SpO_2 _remained stable at T3 and T4**. Data are means +/- standard deviation.

After initial pre-oxygenation and bag valve ventilation (T2), oxygen saturation (SpO_2_) remained stable at T3 and T4 (Figure 4).

## Discussion

In this observational trial, intubation with the Airtraq^® ^optical laryngoscope was feasible, safe and easy to perform in patients undergoing routine cardiac surgery. In all patients, a satisfactory view on the vocal cords could be obtained, independent from BMI and preoperative Mallampati score.

Our results are confirmed by a variety of studies highlighting the usefulness of the Airtraq in tracheal intubation and the management of the difficult airway [11,20,21]. In line with several reports showing a reduction of intubation time and an improvement of intubation conditions, in none of our patients the "intubation score" was ranked worse than grade II. Although this score was originally described to evaluate the view on the vocal cords during direct laryngoscopy, we-due to a lack of better alternatives-used this score also for the indirect laryngoscopy. This is supported by our findings that intubation conditions were subjectively judged as "very good" in the vast majority of patients. Despite the excellent intubation conditions achieved in our trial, few patients still required repeated intubation attempts which were in part due to inherent problems of indirect laryngoscopy (contamination of the optical system). These problems have been reported also for other video-assisted intubation devices [21,22]. In five patients however, the first intubation attempt failed despite a good glottic and upper esophageal aperture view. Failure of tracheal intubation with the Airtraq^® ^in these patients was always associated with the high position of the glottic opening, that is typical for the indirect laryngoscopy but surprising for anesthesiologists not familiar with this technique and hence advancing the tip of the device erroneously too deep. Moreover, the channel design of the Airtraq^® ^directs the tracheal tube initially downwards before it finally ascends. This requires a minimum distance between the exit of the channel and the glottic opening [23]. These problems disappeared after some additional instructions and explanations given by the supervising consultant.

Most probably due to the ease of use, we did not observe any severe complications in our trial. Despite all participating physicians were previously trained in manikin and performed 5 guided Airtraq^® ^intubations in routine patients they could be appraised relatively unfamiliar with the indirect laryngoscopy. In simulated difficult airway settings the Airtraq^® ^device appeared to be superior in the hand of experienced, novice and inexperienced users [15,24]. Hirbayashi demonstrated that the Airtraq^® ^optical laryngoscope reduces both, the time to secure the airway and the incidence of failed tracheal intubation by novice laryngoscopists [25]. Lopez-Negrete underlines that poor viewing conditions occurred less frequent [26]. In patients at an increased risk for difficult tracheal intubation, the Airtraq^® ^reduces the duration of intubation attempts and significantly more patients could be intubated [13]. Moreover, duration of tracheal intubation was shortened and reductions in arterial oxygen saturation were prevented in morbidly obese patients [27,28]. Focusing the group of obese patients (BMI 30-35, n = 28 and BMI > 35, n = 11) in our trail, these data support our finding that no correlation between BMI and the duration of tracheal intubation or SpO_2 _could be observed. Airtraq^® ^has been reported to be an excellent alternative device, managing the expected or unexpected difficult airway in adult and pediatric patients (i.e. ankylosing spondylitis, morbid obesity, rigid collar immobilisation of the cervical spine, caesarean section, trauma and some rare syndromes (Goldenhar, Treacher Collins etc.) [17,18].

The traditional predictors of difficult intubation seem to be less accurate if the Airtraq^® ^is used. Of note, traditional scores predicting difficult intubation were not designed for modern video-laryngoscopes or the Airtraq^® ^, but for devices with a conventional "alignment requiring" as the Macintosh blade.

It is well known that laryngoscopy and tracheal intubation with a conventional Macintosh laryngoscope may cause severe dental injury in 0.04%-0.36% [29,30] and oral tissue trauma in 6.9% [31]. In this trial in 6 patients (4.8%) minor superficial mucosal damages (n = 4) and lip contusions (n = 2) were identified. Two of the mucosal lacerations affected further intubation course and led to a 2^nd ^intubation attempt due to the contamination of the optical system with blood. It remains speculative if laryngoscopy using a Mcintosh laryngoscope would have performed more gently. However, it has been postulated that airway traumata caused by the Airtraq^® ^blade during the initial insertion are less likely to be noticed because of the narrow field view provided [32]. When carefully examining the patients for potential injury, minimal oro-pharyngeal lesions due to difficult pharyngeal insertion were described in up to 5% of lean and 25% of obese patients. Due to the lack of (randomized) controlled or observational trials the exact incidence of dental or oro-pharyngeal lesions in greater number of patients is still unknown. Compared with the low cardiac risk patients in literature not receiving platelet aggregation inhibitors, the use of the Airtraq^® ^may be associated with minor trauma as compared to the Macintosh laryngoscope.

While there is increasing evidence on the usefulness of the Airtraq^® ^in situations of a difficult airway, only few data exist on the easiness and feasibility of the Airtraq^® ^in routine clinical settings and its potential problems and side effects. In this observational trial, focusing on feasibility, cardiovascular response, intubation time and potential side effects of Airtraq^® ^intubation in a cohort of patients undergoing anesthesia for coronary artery bypass grafting were studied.

Due to their underlying disease, these patients are at particular risk to develop perioperative myocardial ischemia due to an imbalance of myocardial oxygen supply and demand as potentially induced by the procedure of endotracheal intubation, which can be associated with an increase in heart rate and arterial blood pressure [33-35]. Singh et al. demonstrated in a comparison of 4 anesthetic induction agents, that stress response on conventional endotracheal intubation was most evident in patients with coronary artery disease when anesthesia was inducted with Etomidate [36]. In our trial anesthesia was inducted with Etomidate. On the contrary although statistically significant, endotracheal intubation with the Airtraq^® ^resulted in a clinically neglectable minimal increase in heart rate, while arterial blood pressure was not affected. Hence, the use of the Airtraq^® ^device allowed to maintain stable hemodynamic conditions. Our results are confirmed by previously published data showing less hemodynamic alteration when endotracheal intubation is performed with the Airtraq^® ^, compared to intubations with the Macintosh laryngoscope [12,27,28]. In a currently published meta-analysis Lu et al. concluded that the Airtraq^® ^produces less hemodynamic stimulation, which may be an advantage in geriatrics or in patients with coronary heart disease or primary hypertension [37]. Connatural hemodynamic stability during indirect laryngoscopy has recently been demonstrated in analogy for the Pentax-AWS^® ^[38].

One possible explanation might be that the use of the Airtraq^® ^requires less force to align the oro-pharyngeal-tracheal axes for tracheal intubation, e. g. the traction force to lift the mandible is reduced and hence induces less pain-mediated activation of the sympathetic nervous system [12,27,28,37].

We acknowledge that our study exhibits various limitations. First, the lack of a control group makes a direct comparison to conventionally used laryngoscopes impossible. Second, all intubations performed with the Airtraq^® ^in this observational trial were performed by 5 anesthetists (consultant and registrar level), each with an experience of > 2000 orotracheal intubations with the conventional Mcintosh blade, advanced skills in the management of the difficult airway and a special Airtraq^® ^practice training. Hence, any conclusions on the performance of the Airtraq device in inexperienced users remain speculative. However, it is probable that anesthetists not familiar with the Airtraq^® ^device or in the early stage of their specialization, success (and complication) rates would have been different [39]. Moreover, because of our limited experience with the Airtraq^® ^in cardiac surgery prior to this study, patients at an obvious risk for difficult intubation were excluded in our observational trial. Despite the increasing evidence of the usefulness of the Airtraq in the management of the difficult airway, it remains to be elucidated whether the use of the Airtraq in cardiac surgical patients with difficult intubation conditions is still associated with the advantages observed in our trial. In addition, due to the lack of validated scores for optical laryngoscopes graduating the view and/or easiness of tracheal intubation (e.g. the Cormack-Lehane intubation grade for the Macintosh blade) we chose a modified score with 4 endpoints, equal to the Cormack-Lehane score, to describe the glottic view achieved during laryngoscopy without the use of the videoscope monitor. In this context the original Cormack-Lehane score is frequently used to demonstrate an improved view when comparing the Macintosh- with optical laryngoscopes [37,40,41]. We have to acknowledge that this score was not designed for this purpose but alternatives are not evaluated yet.

## Conclusion

In conclusion our results demonstrate that routine tracheal intubation with the Airtraq^® ^is feasible, fast and save in high-risk cardiac patients. The use of the Airtraq allowed maintaining a stable hemodynamic situation.

## Competing interests

GS received fees for general lectures in "difficult airway management" from the German Airtraq^® ^distributor Medisize Deutschland GmbH, Neunkirchen-Seelscheid, Germany.

The authors declare that they have no competing interests.

## Authors' contributions

GS and US equally carried out conception and design, anesthesia, interpretation of data and drafted the manuscript. SR participated in interpretation, conducted anesthesia and helped to draft the manuscript. MC critical revised the manuscript and supervised statistical analysis. BF allocated data and conducted anesthesia. RR approved the final version and NZ critical revised the manuscript and coordinated the trial. All authors read and approved the final manuscript.
